# Tunable multi-band terahertz sensor based on graphene plasmonic metasurfaces

**DOI:** 10.1038/s41598-026-36617-9

**Published:** 2026-02-11

**Authors:** Maira Khafagy, AbdelRahman M. Ghanim, Mohamed A. Swillam

**Affiliations:** 1 Department of Physics, School of Science and Engineering, The American University in Cairo, New Cairo, 11835 Egypt; 2 Department of Physics, School, Faculty of Science, Ain Shams University, Cairo, 11566 Egypt

**Keywords:** Surface plasmon resonance, Refractive index, Graphene, Sensing, Materials science, Nanoscience and technology, Optics and photonics, Physics

## Abstract

This study introduces a highly sensitive and tunable plasmonic refractive index sensor based on a novel metal-dielectric-dielectric-metal (MDDM) metasurface architecture operating in the Terahertz (THz) region. The proposed structure consists of a graphene-based fractal pattern integrated with a dielectric layer, a silicon substrate, and a bottom aluminum layer, leveraging commercially available and easy-to-fabricate materials. This multilayer configuration supports strong plasmonic resonances and enhanced absorption, enabling triple-band refractive index sensing with high sensitivities of 10 μm/RIU, 3 μm/RIU, and 2.75 μm/RIU across three distinct modes, surpassing previously reported single- and dual-band plasmonic sensors. Unlike conventional metal-dielectric-metal (MDM) absorbers, the dual-dielectric configuration enhances field localization and supports three distinct resonance modes: a dipolar mode at approximately 7.69 THz, a quadrupolar mode near 25.4 THz, and a hybridized higher-order mode around 30.2 THz. These hybridized resonances arise from the coupled interactions between the central hexagonal graphene core and the concentric double nanorings, producing intense localized electromagnetic “hot spots” that significantly amplify the sensor’s spectral response. The multiple resonances offer improved flexibility for detecting various analytes or extending the sensing range. The proposed MDDM sensor maintains stable and tunable performance under environmental variations, demonstrating significant potential for practical applications in biomedical diagnostics, gas sensing, and glucose monitoring.

## Introduction

The THz frequency range, positioned between the microwave and infrared spectrums, has attracted much attention in the past 20 years, due to its unique interaction with materials. THz waves are ideal for spectroscopy, security screening, biological imaging, and wireless communication due to their extreme sensitivity to rotational transitions and molecular vibrations^1^. THz waves can interact with biological tissues and sensitive materials since they are non-ionizing, in contrast to high-energy radiation like X-rays^2^. The THz spectrum spans from 0.1 to 10 THz, providing a balance between low energy consumption and penetration depth. This makes it particularly suitable for applications such as biomedical imaging, where it enables early disease detection without harmful ionization^3^. Furthermore, THz technology is essential for the remote sensing of dangerous substances^4^, real-time moisture content monitoring in agricultural products^5^, and quality control in semiconductor manufacturing^6^.

Graphene exhibits remarkable plasmonic behavior, especially within the THz and mid-infrared frequency ranges, making it a promising alternative to traditional noble metals for plasmonic applications. Unlike conventional metals, graphene supports tightly confined surface plasmon polaritons (SPPs) that feature longer propagation lengths and significantly lower ohmic losses^7–9^. These plasmons are generated by the collective oscillations of charge carriers near the graphene surface^10,11^. They can be dynamically adjusted through electrostatic gating or chemical doping, permitting real-time control over their frequency and intensity. Moreover, the two-dimensional structure and high electron mobility of graphene enhance the localization and confinement of electromagnetic energy at the nanoscale^12^. This tunability, along with strong field enhancement, opens up a wide range of applications, including modulators, sensors, and photodetectors, especially in the THz range, where conventional materials typically underperform^13,14^. Consequently, graphene-based plasmonics provides a versatile platform for next-generation optoelectronic and nano-photonic devices.

Metamaterials (MMs) are engineered electromagnetic materials designed from periodic arrangements of subwavelength unit cells. These structures can be realized using metals, dielectrics, or advanced materials like graphene^15^. Among the different configurations, the metal–dielectric–metal (MDM) architecture has gained particular attention due to its ability to support strong plasmonic and resonant effects. This structure is especially effective for achieving metamaterial perfect absorbers (MPAs), leveraging unique electromagnetic characteristics such as a negative refractive index (RI)^15–17^. MPAs based on MDM configurations have been explored for a wide array of applications, including chemical and biological^18–20^, photocatalysis, thermal imaging using microbolometers, photothermal conversion, thermal emission control, energy harvesting, and optical switching^21–23^. MDM-based optical resonators are highly responsive to changes in the surrounding environment, making them excellent candidates for RI sensing^24^. Compared to conventional sensor platforms, RI sensors employing MDM metamaterials offer narrowband absorption features and enhanced sensitivity, enabling fast and precise detection. These attributes make them well-suited for use in medical diagnostics, environmental monitoring, and the food industry^25–27^.

One of the primary challenges in designing high-performance biosensors is achieving both high sensitivity and a narrow full width at half maximum (FWHM) simultaneously. Inadequate performance in either parameter often results in poor spectral resolution, reducing the reliability of detection, especially in biomedical applications^15^. To overcome these limitations, plasmonic materials have been widely integrated into biosensing platforms to enhance light–matter interaction and improve detection efficiency. Among these, noble metals such as gold (Au) and silver (Ag) have been extensively used due to their excellent plasmonic properties and ability to support strong surface plasmon resonances, leading to enhanced sensitivity and detection limits^28–30^. However, the high cost and limited tunability of these metals remain significant drawbacks, particularly for large-scale or disposable biosensor applications. As a result, there is growing interest in exploring more cost-effective alternatives that do not compromise performance.

This study introduces a multilayer tunable plasmonic graphene RI sensor using metal-dielectric-metal (MDM) in the THz region. Finite-Difference Time-Domain (FDTD) simulations confirm that these modes exhibit linear and highly sensitive frequency shifts under refractive index variations, achieving sensitivity of up to 1000 nm/RIU. Operating in the THz range, the proposed sensor exhibits multiband resonance spectra with high sensitivities, highlighting its strong potential for high-performance biosensing applications. The proposed sensor is adaptable to varying conditions, ensuring stable performance and demonstrating the potential of THz-based plasmonic devices in advanced sensing technologies.

## Materials and methods

Graphene’s surface conductivity can be derived using the Kubo formula, which accounts for intraband and interband contributions, and it can be expressed as^31,32^:

1 $$\:\sigma\:\left(\omega\:\right)=\:{\sigma\:}_{intra}\left(\omega\:\right)+{\sigma\:}_{inter}\left(\omega\:\right)$$

2 $$\:\sigma\:\left(\omega\:\right)=\frac{2{e}^{2}{k}_{B}T}{\pi\:\hslash\:(\omega\:+j{\tau\:}^{-1})}\mathrm{ln}[2\mathrm{cosh}\left(\frac{{E}_{F}}{2{k}_{B}T}\right)]+\frac{{e}^{2}}{4\hslash\:}[\frac{1}{2}+\frac{1}{\pi\:}\mathrm{arctan}\left(\frac{\hslash\:\omega\:-2{E}_{F}}{2{k}_{B}T}\right)-\frac{1}{2\pi\:}\mathrm{ln}\frac{{\left(\hslash\:\omega\:+2{E}_{F}\right)}^{2}}{{\left(\hslash\:\omega\:-2{E}_{F}\right)}^{2}+4{\left({k}_{B}T\right)}^{2}}]$$

For the lower THz frequency range, the effects of interband transitions and optical phonon emissions are less pronounced and can be disregarded. This approximation holds particularly well when the Fermi level ($\:{E}_{F}$) is higher than the thermal energy ($\:{K}_{B}T$), ensuring that the majority of electron transitions occur within the conduction band^33^. When performing calculations in practice, a temperature of 300 K is a reasonable approximation for room-temperature experiments.

Assuming a highly doped graphene structure, the Pauli exclusion principle becomes applicable. In this regime, the surface conductivity of graphene can be approximated using the intraband Drude model. This approach provides a simplified yet accurate representation of the material’s response to an external electromagnetic field. The Drude model expresses conductivity as^34^:

3 $$\:\sigma\:\left(\omega\:\right)=\:\frac{{e}^{2}}{\pi\:{\hbar}^{2}}\:\frac{i}{\omega\:+i/\tau\:}$$

where, $\:\omega\:$ is the angular frequency of the incident wave, $\:e$ is the elementary charge, $\:\hbar=h/2\pi\:$, is the reduced Planck constant, and $\:\tau\:$ is the relaxation time, which accounts for carrier scattering processes. The relaxation time and Fermi energy can be further expressed as

4 $$\:\tau\:=\:\mu\:\frac{\hbar\sqrt{\pi\:\:\left|{n}_{g}\right|}}{e{v}_{F}}$$

5 $$\:{E}_{F}=\hbar{v}_{F}\:\sqrt{\pi\:\:\left|{n}_{g}\right|}$$

$\:{n}_{g}$,$\:{v}_{F}={10}^{6}m/s$ and $\:\mu\:=1000\:c{m}^{2}/\:V.s$ are the graphene doping level, the velocity of the graphene Fermi, and the measured $\:{d}_{c}$ mobility, respectively^35^.

**Fig. 1:**
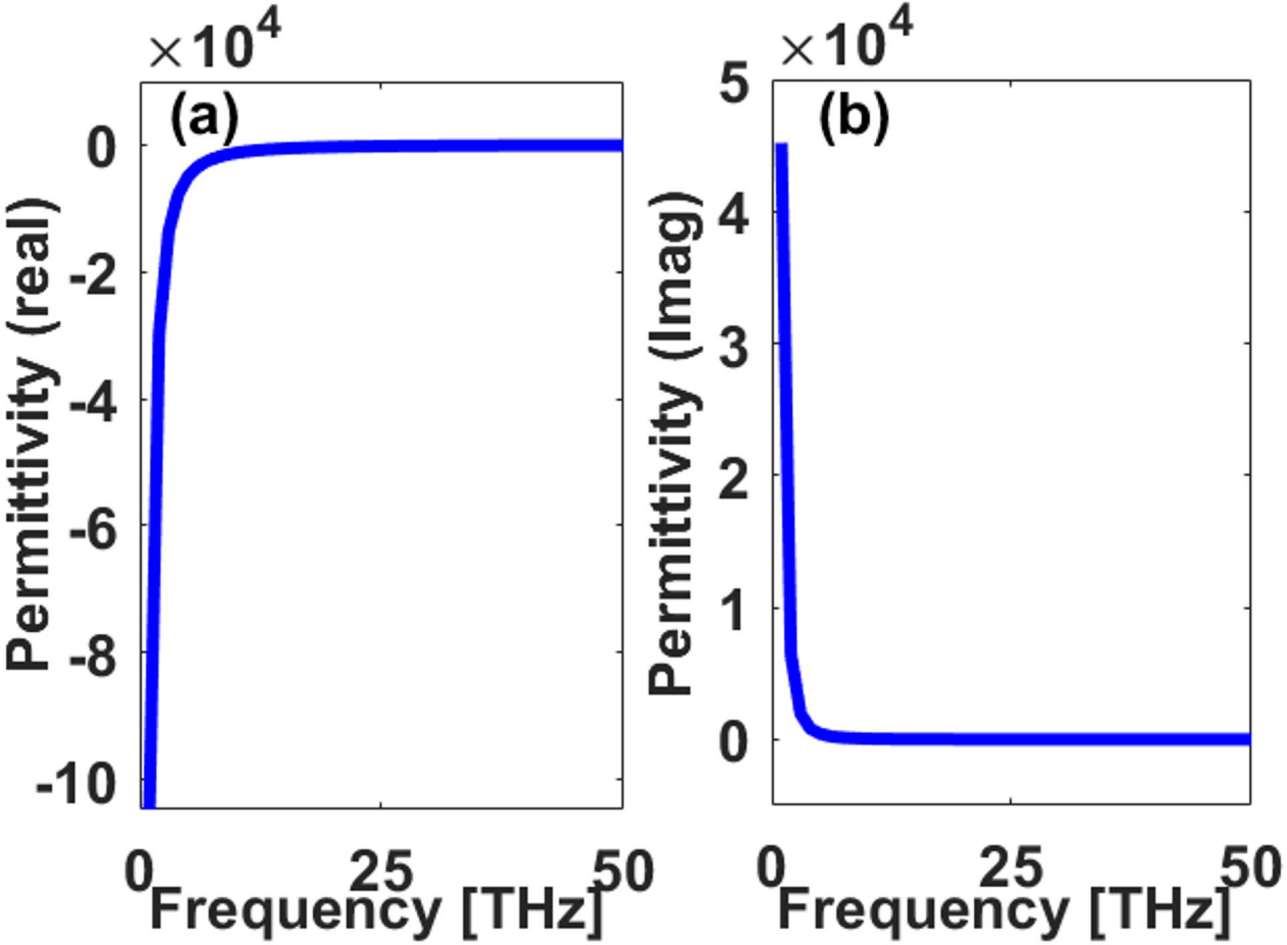
Permittivity of graphene as a function of frequency in the terahertz region: (**a**) Real part of the permittivity (Re(*ε*)) showing negative values that enable plasmonic resonance; (**b**) Imaginary part of the permittivity (Im(*ε*)) indicating material losses.

The generation of surface plasmons (coherent oscillations of free charge carriers at the interface between a conductor and a dielectric) is closely related to the real part of the material’s permittivity (Re(*ε*))^36^. For material to support plasmonic modes, the real part of its permittivity must be negative, allowing for the confinement of electromagnetic fields at the interface. As shown in Fig. 1a, the real part of graphene’s permittivity becomes increasingly negative as the wavelength increases within the terahertz regime. This behavior confirms that graphene can support plasmonic excitations at these wavelengths. The negative values of Re(*ε*) lead to strong field confinement near the surface of the material, a critical factor for enhancing the sensitivity of plasmonic biosensors. In contrast, Fig. 1b displays the imaginary part of the permittivity (Im(ε)), which is associated with the material’s absorption losses. While a high Im(*ε*) can result in increased energy dissipation, the balance between confinement (enabled by negative Re(*ε*)) and losses (dictated by Im(*ε*)) determines the efficiency of plasmon propagation and sensor performance. In the case of graphene, the relatively low imaginary component in the lower terahertz range suggests that the material can support low loss plasmonic modes suitable for sensing applications.

Following the graphene layer, the proposed MDDM sensor incorporates a carefully engineered dielectric stack and a metallic ground plane to achieve strong and tunable resonance behavior. A dielectric layer with a refractive index (RI) of ~ 2.0, corresponding to silicon nitride (Si_3_N_4_)^37^, is used to control resonance tuning, electromagnetic field confinement, and the effective optical path length, with its thickness optimized for high sensitivity and selectivity in triple-band operation. An additional silicon layer is placed above this dielectric to enhance field confinement and RI contrast further, resulting in sharper and stronger resonance peaks while providing greater flexibility in frequency tuning with minimal loss in the THz regime. The structure is completed with an aluminum ground plane, whose strongly negative real permittivity and relatively low imaginary component in the terahertz range support surface plasmon polaritons, improve electromagnetic field confinement, reduce losses, and enhance light trapping, thereby sharpening resonance features essential for high-performance multi-band biosensing.

## Biosensor design

###  Structure design of the proposed sensor

**Fig. 2:**
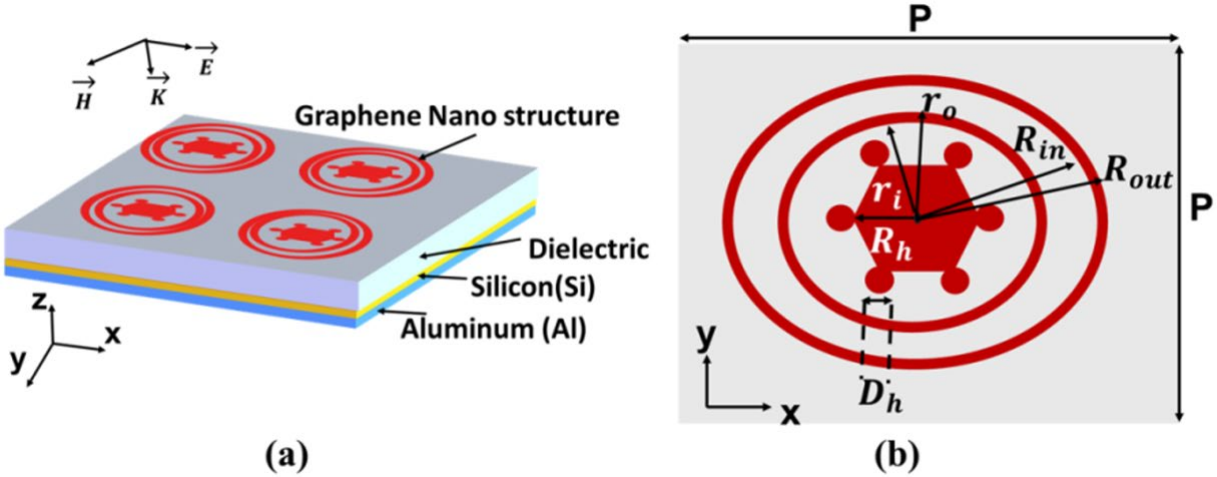
(**a**) Multi-layer-dimensional illustration of the proposed sensor, (**b**) Orthographic top view of the unit cell.

The investigated structure is a multilayer double nanoring-strip system composed of periodic arrays of graphene nanoring-strips patterned on an MDM substrate, as shown in Fig. 2. The nanostructured graphene is mounted on the sheet of a dielectric layer with RI of 2, followed by an intermediate (Si) layer, and a bottom Al substrate. The dielectric and Si layers serve as dielectric spacers, while the graphene and aluminum function as metallic components, forming an MDDM architecture. The presence of the Si layer plays a crucial role in enhancing light confinement and field localization, thereby significantly improving the optical response of the structure. The combination of the double nanoring and the hexagonal core structure further improves the plasmonic response, making the design highly suitable for advanced applications such as photodetectors, filters, and optical sensors. The arrays are arranged with a fixed period (*P*) of 300 nm, the thickness of the dielectric is 1.5 μm, and the thickness (*t*) of the graphene is 1 nm. Figure 2a shows the structure of the multilayer configuration begins with a top layer of graphene double nanoring strips. The nanostructure consists of a central hexagonal element surrounded by a concentric double nanoring. The hexagon hosts six symmetrically positioned circular holes, arranged around a central axis to form a regular hexagonal pattern. Each hole has a diameter $\:{D}_{h}$, and they are positioned at a radial distance $\:{R}_{h}$ from the center of the hexagon. The inner and outer radii of the nanoring are represented by $\:{r}_{i}$ and $\:{r}_{o}$, respectively. Additionally, the graphene rings extend further with radii $\:{R}_{in}$ and $\:{R}_{out}$, forming a double nanoring-strip configuration as shown in Fig. 2b. This configuration enhances the tunability of the structure’s optical and plasmonic characteristics through the coupling effects between the hexagonal core and the encompassing double nanoring. The positions of the nanorod centers, which are symmetrically distributed around the central hexagon, are precisely determined to ensure that the outer edges of the nanorods are in direct contact with the hexagon’s vertices. This spatial arrangement is achieved by employing polar coordinates and applying the geometric relations outlined in Eqs. (4) and (5)^30^.

6 $$\:{r}_{n}=\:{R}_{h}+\frac{{D}_{h}}{2}$$

7 $$\:{\theta\:}_{n}=\frac{\pi\:}{3}\:.\:n$$

where *n* = 0, 1, 2, 3, 4 and 5.

## Numerical modeling

In this study, the finite-difference time-domain (FDTD) technique was utilized to analyze the spectral response of the graphene nanoring-strip structures. The transmission spectrum and electric field distributions were simulated using the FDTD software^38^. Periodic boundary conditions were applied along the *x* and *y* axes, while perfectly matched layer (PML) boundaries were used along the *z*-direction for both ( $\:{\mathrm{z}}_{\mathrm{m}\mathrm{i}\mathrm{n}}$ and $\:{\mathrm{z}}_{\mathrm{m}\mathrm{a}\mathrm{x}}$) to model the propagation of the incident light accurately. A plane wave source was launched to illuminate the entire graphene array, providing a comprehensive representation of its optical response. The source emits an electromagnetic wave within the frequency range of 1 THz to 40 THz, propagating toward the material. To achieve high-resolution accuracy in key regions of the structure, specific mesh override settings were applied. As the incident wave interacts with the structure, a portion of it is transmitted through the material, while the rest is reflected.

**Fig. 3:**
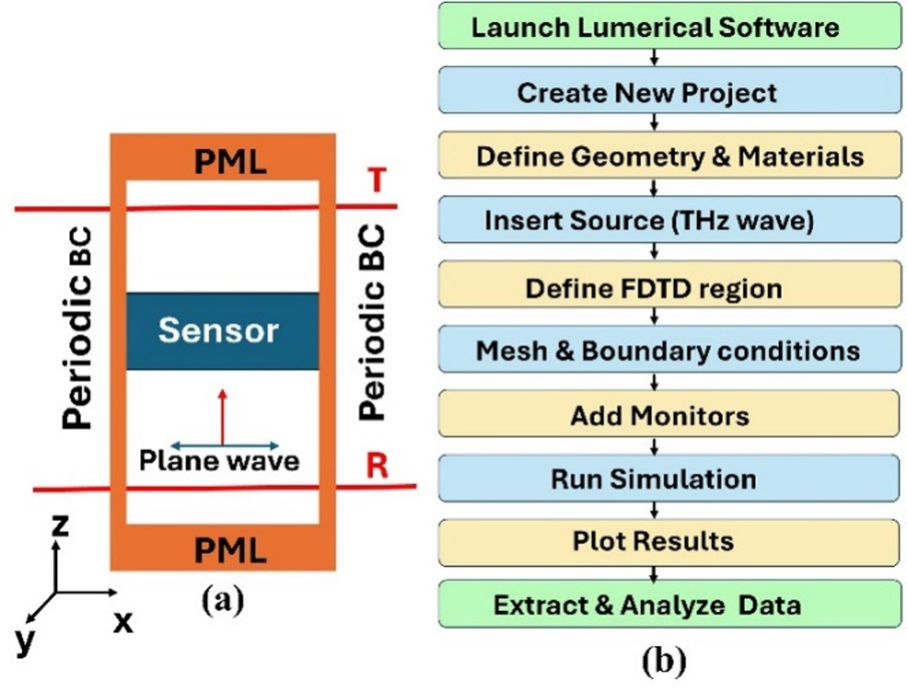
(**a**) 3D schematic of the FDTD simulation setup used to model the proposed graphene-based MDDM metasurface unit cell. Periodic boundary conditions are applied along the *x*- and *y*-directions, while PMLs are used along the *z*-direction. (**b**) Workflow diagram illustrating the numerical simulation procedure in Lumerical FDTD.

Two FDTD power monitors are strategically placed to capture and compute the transmittance (T) and reflectance (R) spectra, as illustrated in Fig. 3a. The absorption spectrum (A) is then derived using the calculated transmittance and reflectance values according to the following relation^39^:

8 $$\:A=1-R-T$$

The sensitivity (*S*) of the sensor is defined based on its response to variations in the refractive index, as the structure operates based on the frequency shifts. The following relation can express the sensitivity^40^

9 $$\:S=\:\frac{\varDelta\:{f}_{r}}{\varDelta\:n}$$

where, $\:\varDelta\:{f}_{r}\:$represents the shift in the resonance wavelength observed in the absorption spectrum, and $\:\varDelta\:n$ denotes the corresponding change in the RI of the analyte or surrounding medium. This metric is crucial for assessing the sensor’s ability to detect subtle environmental or material variations. The performance of the sensor is evaluated by calculating the Q-factor and figure of merit (FOM). These parameters provide more information about the sensor’s performance, such as resolution and detection accuracy of plasmonic sensors. The Q-factor, which measures the sharpness of a resonance, is defined as^41^:

10 $$\:Q=\frac{{f}_{r}}{\varDelta\:f}$$

where, $\:{f}_{r}$ is the center resonance frequency and $\:\varDelta\:f$ is the FWHM of the resonance peak. The figure of merit (FOM), which evaluates the trade-off between sensitivity and linewidth, is expressed as^41^:

11 $$\:FOM=\frac{S}{\varDelta\:{f}_{r}}$$

Figure 3b illustrates the comprehensive workflow for simulating a THz sensor using Lumerical software. The process begins by launching the software and creating a new project or simulation file. Geometry and material properties, such as graphene, dielectric layers, silicon, and aluminum, were defined to build the sensor structure. A suitable excitation source, typically a THz plane wave, is inserted to stimulate the device. Monitors are added to record various fields, power, and index data during the simulation. Subsequently, analysis groups and post-processing settings are configured to extract and plot the absorption characteristics. After performing the time domain simulation, the resulting data, including field distributions and resonance shifts, are extracted and analyzed. Finally, results are plotted to evaluate performance metrics such as refractive index sensitivity and resonance shifts, before exporting the data for further interpretation. This systematic approach ensures accurate modeling and analysis of the sensor’s optical behavior.

**Fig. 4:**
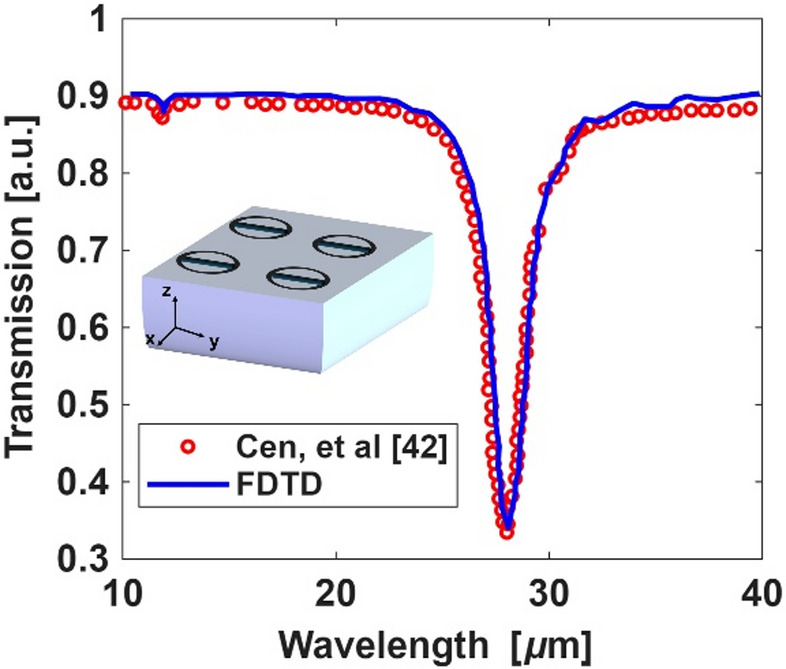
The transmission spectrum of the nanoring graphene sensor^42^ and the FDTD simulation.

In order to validate the accuracy of the 3D FDTD simulation results used in our study, we replicate the structure and results reported in^42^, where a tunable plasmonic RI sensor based on nanoring graphene arrays is presented^42^. The structure consists of a periodic array of nanoring graphene elements embedded between a semi-infinite substrate (*n*_suβ_) and a sensing medium (*n*_me_). The periodicity of the array is fixed at 300 nm, with a nanoring width of 30 nm and a graphene thickness of 1 nm. The strip has a width of 30 nm and a variable length, initially set to 180 nm. Using the FDTD method, we simulate the transmission response of this structure and analyze the shift in resonance with variations in the strip length and refractive index of the sensing medium. As shown in Fig. 4, our simulation results are compared to those reported in^42^ and a strong agreement is observed.

## Sensitivity estimation

### Evolution of graphene geometries in the proposed sensor design

**Fig. 5:**
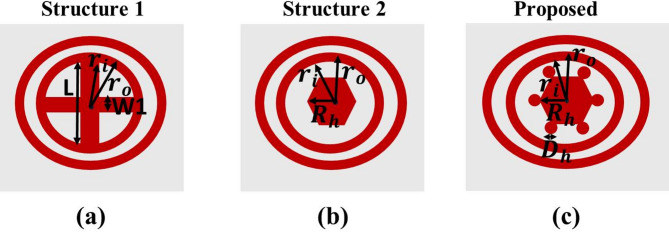
Evolution of the graphene unit cell geometry employed in the proposed MDDM sensor. (**a**) The initial design with a circular graphene resonator of outer radius $\:{r}_{0}$ and a cross-shaped slot defined by length *L* and width $\:{w}_{1}$ (**b**) Intermediate structure formed by replacing the central circular region with a hexagonal graphene core of radius $\:{R}_{h}$, surrounded by concentric rings with radii $\:{r}_{i}$ and $\:{r}_{0}$. (**c**) Final optimized unit cell featuring six symmetrically placed circular graphene patches of radius $\:{R}_{h}$ at the hexagon, separated by a distance $\:{D}_{h}$, enabling enhanced plasmonic coupling and multi-band resonance.

To optimize the electromagnetic response and sensing capabilities of the proposed sensor, various geometrical configurations of the graphene layer were explored, as illustrated in Fig. 5. The evolution began with a basic circular resonator structure with an embedded cross-shaped slot Fig. 5a, intended to introduce polarization-independent behavior and strong resonance confinement. In the next stage, as illustrated in Fig. 5b, the central region was modified to incorporate a hexagonal shape, which aligns with the intrinsic symmetry of graphene’s atomic lattice^43^. This adjustment is expected to potentially enhance charge distribution and strengthen coupling with the incident terahertz waves^44^. Finally, additional circular patches were added to the vertices of the hexagon in the third configuration, as shown in Fig. 5c, creating localized surface plasmon hotspots and increasing the effective interaction area. This gradual design refinement was intended to improve field confinement and boost the sensitivity of the structure for sensor applications.

### Geometrical parameters for the graphene structure

The geometrical parameters used in each configuration are summarized in Table 1. These parameters were carefully selected to maintain nanoscale dimensions suitable for infrared and THz applications while enabling potential electrical tunability.

**Table 1:** The geometrical parameters for the different shapes of the graphene layer.

Structure	Parameter	Value
Circular resonator with a cross slot	$\:{W}_{1}$	24 nm
	*L*	180 nm.
	$\:{r}_{i}$	12 nm
	$\:{r}_{o}$	14 nm
Concentric rings with a central hexagon	$\:{R}_{h}$	80 nm
	$\:{r}_{i}$	12 nm
	$\:{r}_{o}$	14 nm
Enhanced hexagonal structure with peripheral patches	$\:{D}_{h}$	40 nm
	$\:{R}_{h}$	80 nm
	$\:{r}_{i}$	12 nm
	$\:{r}_{o}$	14 nm

### Performance evaluation of each graphene structure

**Fig. 6:**
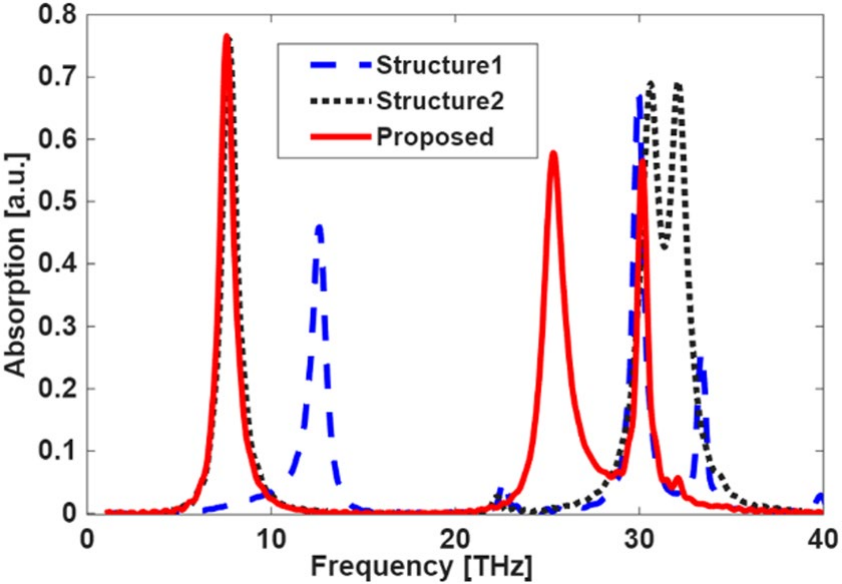
Absorption spectra as a function of frequency for the three graphene structures.

To evaluate the impact of geometrical modifications on the sensor’s absorption characteristics, the frequency response of the three graphene-based configurations presented in Fig. 5 was analyzed, with the results shown in Fig. 6. The configuration corresponding to Structure-1 (blue dotted line), based on the basic circular resonator with a cross-slot, shows three distinct but relatively narrow absorption peaks centered at approximately 12.6 THz, 30 THz, and 33.4 THz, with corresponding absorption magnitudes of 0.42, 0.65, and 0.23. Structure-2 (black dashed line), incorporating a central hexagon, introduces three resonant modes with improved performance that appear around 10.8 THz, 30.7 THz, and 32.1 THz, reaching absorption values of about 0.56, 0.63, and 0.63. As shown in Fig. 6, the last two resonance modes exhibit noticeable spectral overlap, leading to mode interference within the higher frequency region. This coupling effect causes the individual resonance peaks to merge partially, forming a broadened composite peak. However, the most significant enhancement is observed in the proposed design (red solid line), which integrates additional circular patches to the hexagon vertices, exhibits three dominant resonances located near 7.69 THz and 25.4 THz, and 30.2 THz, and achieves peak absorption values of approximately 0.76 and 0.57, and 0.55, respectively. This final configuration achieves stronger absorption peaks, indicating superior light–matter interaction and enhanced sensing performance. The improved response confirms that the added features contribute to better field confinement and multi-resonant behavior, making the proposed design the most effective among the tested variants.

Although the third and second resonance modes of the proposed MDDM structure appear at 25.4 THz and 30.2 THz, these frequencies remain highly relevant for practical sensing applications. While the classical “terahertz gap” is often defined as 0.1–10 THz, recent advances in metasurfaces, near-IR/THz hybrid photonics, and quantum-cascade light sources have significantly extended the usable operating window into the upper-terahertz and far-infrared regime (10–40 THz). Many biomolecular vibrational signatures have critical applications in fields such as biosensing^45^, nano antenna sensing^43^ and Filament Plasma^46^.

**Fig. 7:**
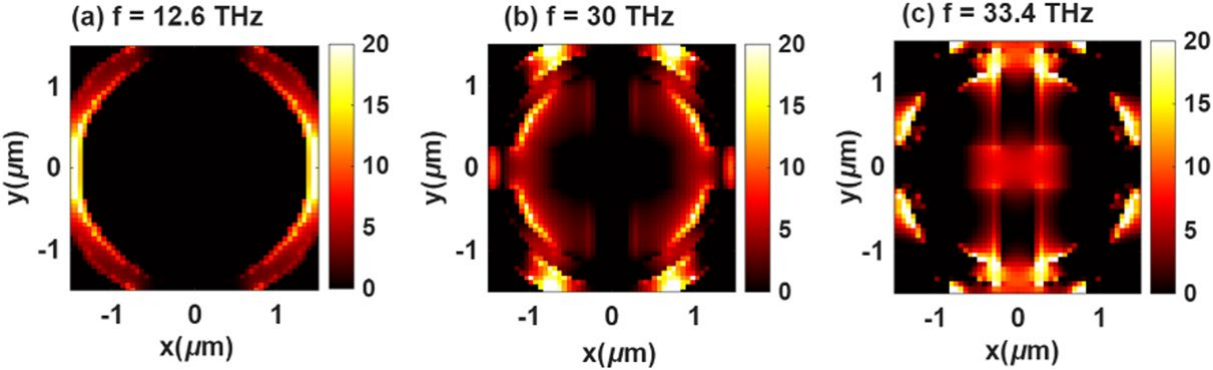
Electric field of structure-1: analysis of the three main absorption peaks (**a**) E-field at *f* = 12.6 THz, (**b**) E-field at *f* = 30 THz, (**c**) E-field at *f* = 33.4 THz.

The electromagnetic behavior of Structure-1, based on a circular resonator with a cross-slot aperture, reveals three distinct resonant modes that correspond to different field confinement mechanisms and current distributions within the metasurface. The first resonance, observed at 12.6 THz (Fig. 7a), is primarily associated with a fundamental dipolar mode, where the electric field is symmetrically concentrated along the outer circular edges, forming a closed current loop. This mode arises from strong capacitive coupling between the opposite sides of the resonator, leading to localized surface plasmon excitation^47^. The second resonance, at 30 THz (Fig. 7b), corresponds to a quadrupolar resonance, characterized by field enhancement at the four arms of the cross-slot. Here, opposite lobes carry anti-parallel surface currents, generating a more complex charge distribution that increases the interaction between the slot gaps and the circular boundary. The third resonance, appearing at 33.4 THz (Fig. 7c), can be attributed to a higher-order hybrid mode, where coupling between the circular ring and the cross-slot induces asymmetric charge oscillations. This produces multiple localized hot spots around the slot terminals and inner ring boundaries, indicating enhanced field confinement and strong plasmonic coupling.

**Fig. 8:**
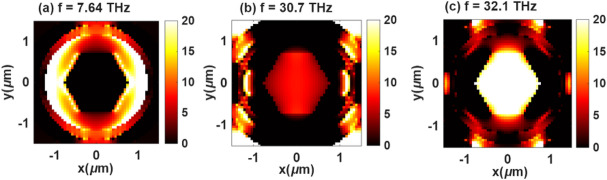
Electric field distributions of structure-2 at (**a**) *f* = 7.64 THz, (**b**) *f* = 30.7 THz, (**c**) *f* = 32.1 THz.

The resonance behavior of Structure-2 reveals three distinct modes corresponding to different electromagnetic coupling mechanisms within the hybrid hexagonal-ring geometry. Mode-1, occurring at approximately 7.64 THz, exhibits a dipolar resonance that is primarily corner-localized, where the electric field is concentrated near the hexagonal vertices, as shown in Fig. 8a. This behavior indicates strong surface charge accumulation at the edges, resulting in efficient light confinement and enhanced local field intensity. Mode-2, observed at around 30.7 THz (Fig. 8b), corresponds to a hybrid ring-hexagon coupled mode. In this regime, both the outer ring and the inner hexagonal frame participate in resonance, forming a coupled plasmonic state that enhances the overlap between the electromagnetic fields and the active region. Finally, Mode-3 (Fig. 8c), appearing near 32.1 THz, represents an anti-bonding (interference) mode, where opposing charge oscillations between the inner and outer layers produce destructive interference in certain regions and field reinforcement in others. This mode is characterized by strong confinement at the inner boundaries and high-order field distributions.

**Fig. 9:**
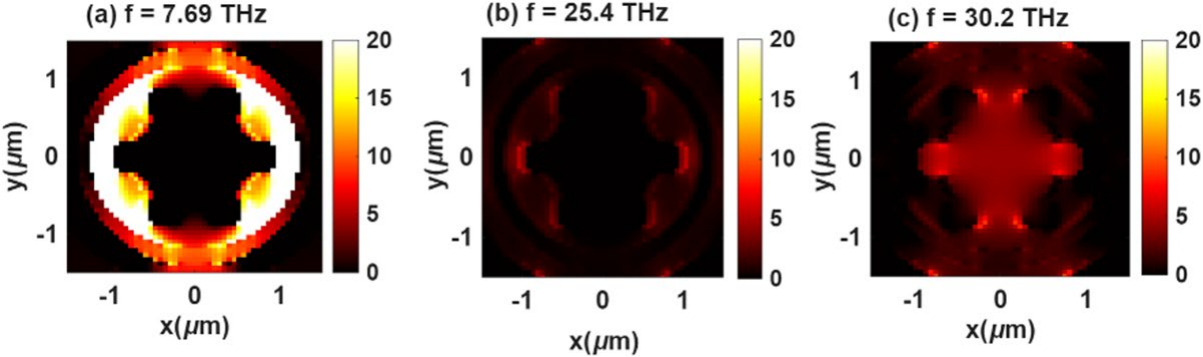
Electric field profiles of the proposed structure at (**a**) *f* = 7.69 THz, (**b**) *f* = 25.4 THz, (**c**) *f* = 30.2 THz.

The proposed structure, which integrates additional circular patches at the vertices of the central hexagon, also exhibits three dominant resonant modes that illustrate a clear transition from localized to hybridized plasmonic behavior. Mode-1, appearing at approximately 7.69 THz, corresponds to a fundamental dipolar resonance as shown in Fig. 9a. In this mode, the electric field is strongly confined around the outer circular patches and edges of the hexagonal frame, indicating that the added patches effectively extend the current path and enhance charge oscillations. This results in pronounced field localization and stronger light–matter coupling compared to the previous designs. Mode-2, located near 25.4 THz (Fig. 9b), represents a quadrupolar (higher-order) resonance that arises from the coupling between adjacent circular patches and the central hexagonal body. The field distribution in this mode shows alternating charge regions, signifying the formation of multiple hot spots that enhance absorption through constructive interference of localized surface plasmons. Finally, Mode-3 (Fig. 9c), observed around 30.2 THz, which corresponds to a hybridized plasmonic mode, where both the inner hexagon and outer patches oscillate collectively. This mode exhibits strong field confinement at both the center and the periphery, reflecting an efficient coupling between the dipolar and higher-order resonances.

## Effect of plasmonic substrate material selection on sensor characteristics

**Fig. 10:**
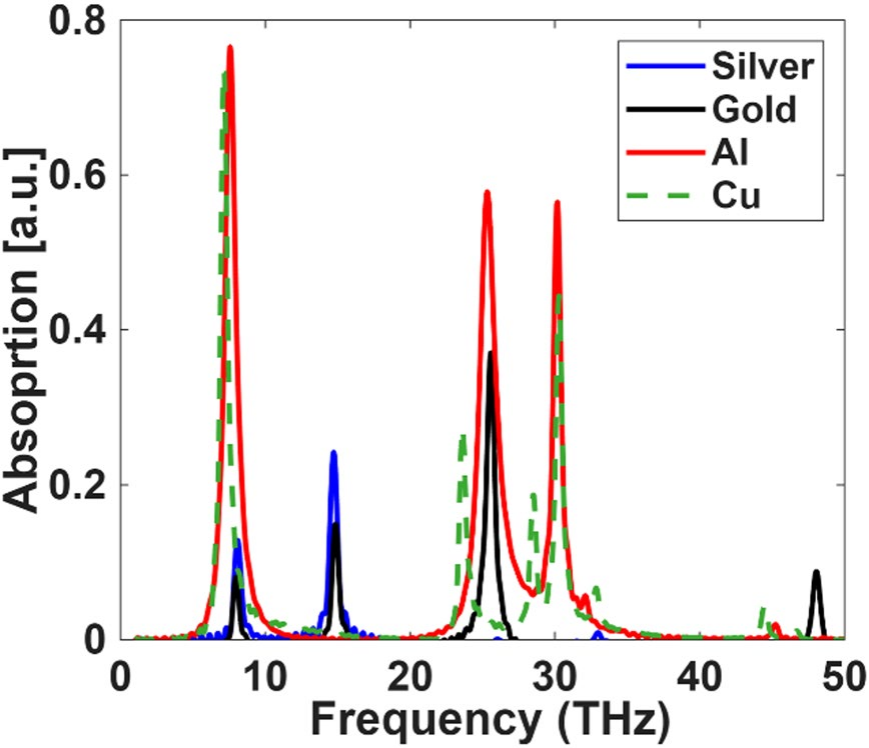
Absorption spectra of the metasurface structure using different conductive materials (Silver, Gold, Aluminum, and Copper) across the terahertz frequency range.

In our proposed biosensor structure, we focus on achieving an optimal balance between performance and cost-effectiveness by selecting low-cost plasmonic materials for the substrate layer. This layer plays a critical role in enhancing both the field confinement and the sensitivity of the sensor. To evaluate the influence of substrate material on biosensing performance, we investigated different conductive materials and their corresponding absorption characteristics across the THz frequency range. The analysis included common choices such as Al, silver, gold, and copper, as shown in Fig. 10.

Figure 10 presents the absorption spectrum obtained for the proposed structure when varying the substrate material. The figure illustrates the absorbance spectra of four metals, silver (Ag), gold (Au), aluminum (Al), and copper (Cu), as a function of frequency. Each metal exhibits two primary resonant peaks. The first resonance, located between 8 and 10 THz, shows that aluminum (red) and gold (black) achieve the highest absorbance values (approximately 0.7–0.75), while silver (blue) and copper (green) display slightly weaker but still noticeable responses. The second dominant resonance occurs near 28–32 THz, where Al, Au, and Cu present strong, narrow peaks with intensities ranging from 0.5 to 0.7. In this region, copper and gold exhibit nearly resonant magnitudes, though copper shows a broader spectral tail. Silver, in contrast, demonstrates a weaker absorption response at higher frequencies. Overall, gold and copper share similar spectral behavior, while aluminum stands out for its more distinct and higher-amplitude resonant characteristics.

## Effect of silicon layer on sensor characteristics

**Fig. 11:**
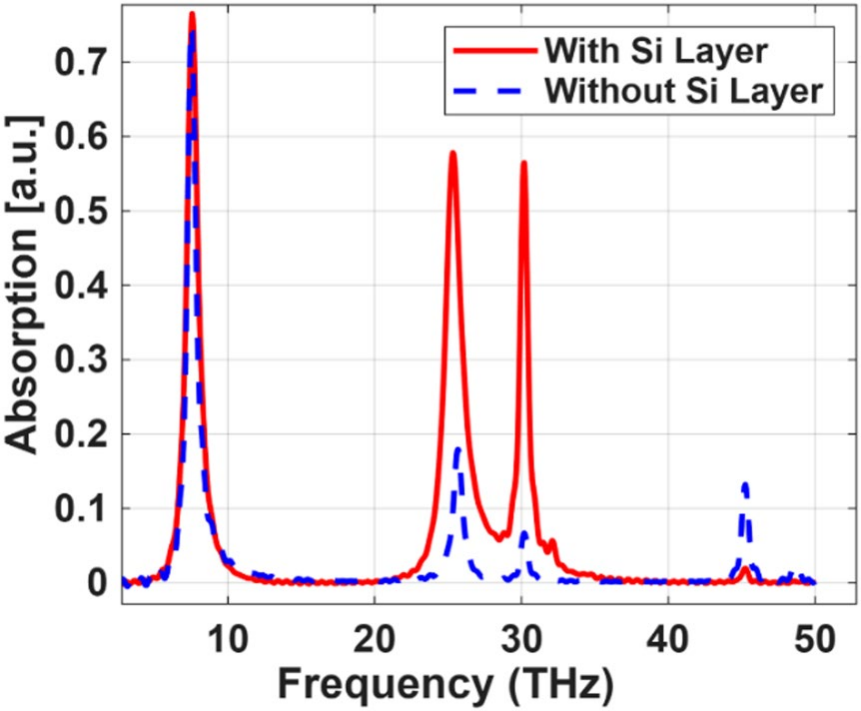
The absorption spectra across the frequency range, comparing the results obtained with and without adding a silicon layer to the dielectric material of the metasurface design.

To enhance the electromagnetic response and sensing capabilities of the proposed multilayer structure, a high-resistivity Si layer was strategically introduced between the dielectric and the substrate. Si is widely recognized for its excellent dielectric properties, low loss tangent in the terahertz regime, and high RI (~ 3.4 in the THz range), making it an ideal material for manipulating electromagnetic wave propagation. The integration of the Si layer plays a critical role in modifying the effective impedance and field distribution within the structure, leading to stronger field confinement near the resonant elements. This results in significantly enhanced absorption peaks and sharper resonance features, as observed in the comparison between structures with and without the Si layer, as shown in Fig. 11. Furthermore, the presence of the silicon layer increases the effective interaction volume between the incident THz waves and the active sensing region, which is essential for improving sensitivity to RI changes or analyte presence in sensing applications. The enhanced absorption observed at multiple resonant frequencies confirms the role of the Si layer in supporting multiple resonance modes, which can be tuned for multi-frequency detection. As illustrated in Fig. 11, the proposed sensor demonstrates improved absorption in the THz range. This enhancement is attributed to the increased interaction between the incident THz waves and the engineered structure of the sensor^48^.

**Fig. 12:**
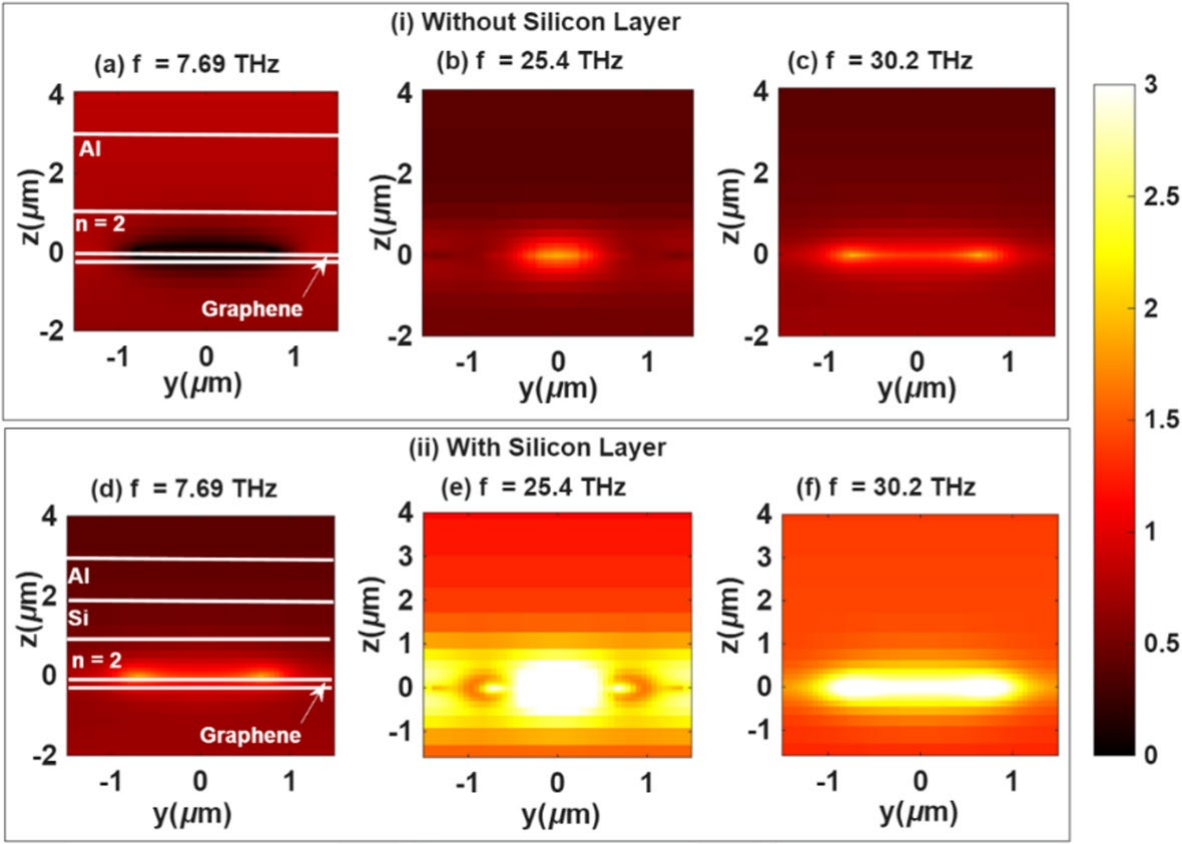
Side view of the electric field distribution, (i) without silicon at (**a**) *f* = 7.69 THz, (**b**) *f* = 25.4 THz, (**c**) *f* = 30.2 THz, and (ii) with silicon at (**d**) *f* = 7.69 THz, (**e**) *f* = 25.4 THz, (**f**) *f* = 30.2 THz.

As shown in Fig. 12, the side-view electric field distribution highlights the crucial role of the silicon layer in enhancing absorption within the terahertz range. A comparison between subfigures (a), (b), and (c) (without silicon) and their corresponding cases (d), (e), and (f) (with silicon) reveals a substantial increase in electric field intensity in the area with the silicon layer. This enhancement signifies stronger confinement and interaction of the terahertz field with the sensor’s active structure. Specifically, at frequencies of 25.4 THz and 30.2 THz, the presence of silicon introduces additional free-carrier absorption mechanisms, which amplify the electric field intensity and improve absorption efficiency. This behavior underscores the crucial role of the silicon layer in facilitating efficient terahertz energy absorption, thereby enhancing the sensor’s optimized performance. The strong sensing performance of the proposed MDDM metasurface can be directly attributed to the pronounced electromagnetic field confinement. In particular, the electric field is localized at the graphene–dielectric interfaces and within the nanoring–cavity regions, indicating plasmon coupling and enhanced light–matter interaction. This confinement results in pronounced resonance features with narrower linewidths, which in turn enhances the Q-factor and figure of merit (FOM).

## Structure optimization

The dimensional parameters of the proposed MDDM sensor were optimized to enhance sensing performance. The optimization process was carried out using a conventional parametric variation approach. For each parameter set, we evaluated the resonance frequency, sensitivity, FWHM, Q-factor, and FOM. The optimal configuration was chosen by maximizing sensitivity while ensuring narrow resonance linewidths. While recent studies have demonstrated the effectiveness of machine learning assisted optimization techniques for metasurface and nanophotonic devices^49^.

### Optimization of the fermi energy

**Fig. 13:**
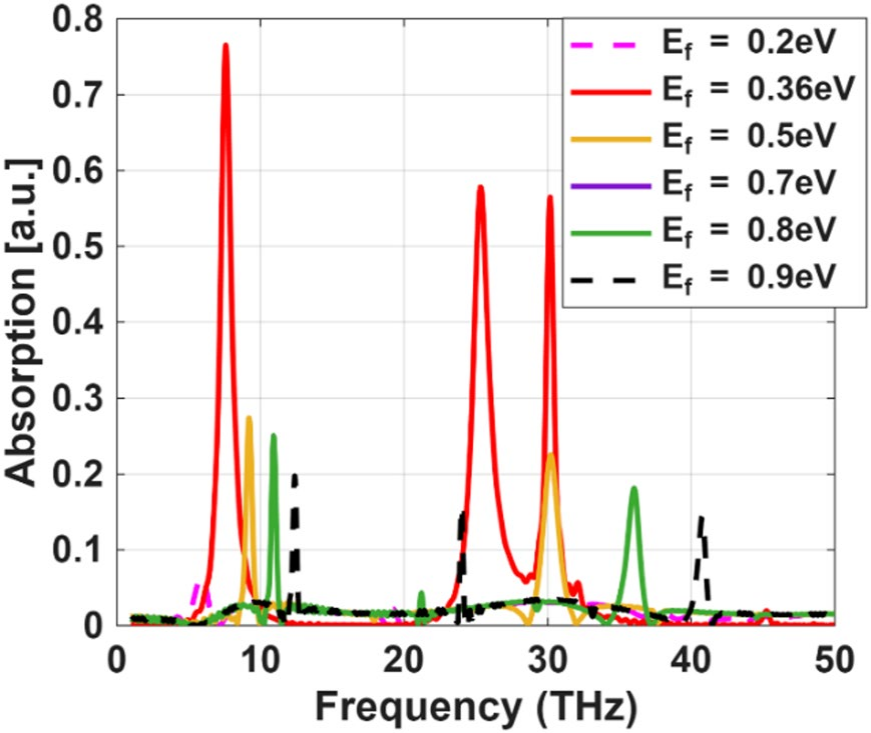
Absorption spectra of the proposed graphene-based terahertz sensor for different Fermi energy values varying from 0.2 eV to 0.9 eV.

Figure 13 illustrates the absorption spectra of the proposed graphene-based terahertz sensor as a function of the Fermi level ($\:{E}_{f}$) varied from 0.2 to 0.9 eV. It is evident that tuning $\:{E}_{f}$ significantly influences the plasmonic response, leading to pronounced changes in resonance amplitude, bandwidth, and spectral position across the investigated frequency range. At lower Fermi levels (e.g., 0.2 eV), the absorption peaks are relatively weak and broad, indicating limited plasmon excitation and reduced electromagnetic confinement. As $\:{E}_{f}$ increases, the resonances become stronger and more distinct due to enhanced carrier concentration in graphene, which strengthens plasmon coupling at the graphene–dielectric interface. Notably, $\:{E}_{f}=0.36$eV (proposed value) yields the highest absorption amplitudes and the sharpest resonance features across all operating bands, reflecting optimal confinement and minimal damping. Further increasing $\:{E}_{f}$ beyond this value results in reduced peak intensity or spectral shifts, suggesting increased losses or overdamping effects.

### Optimization of the aluminum layer

**Fig. 14:**
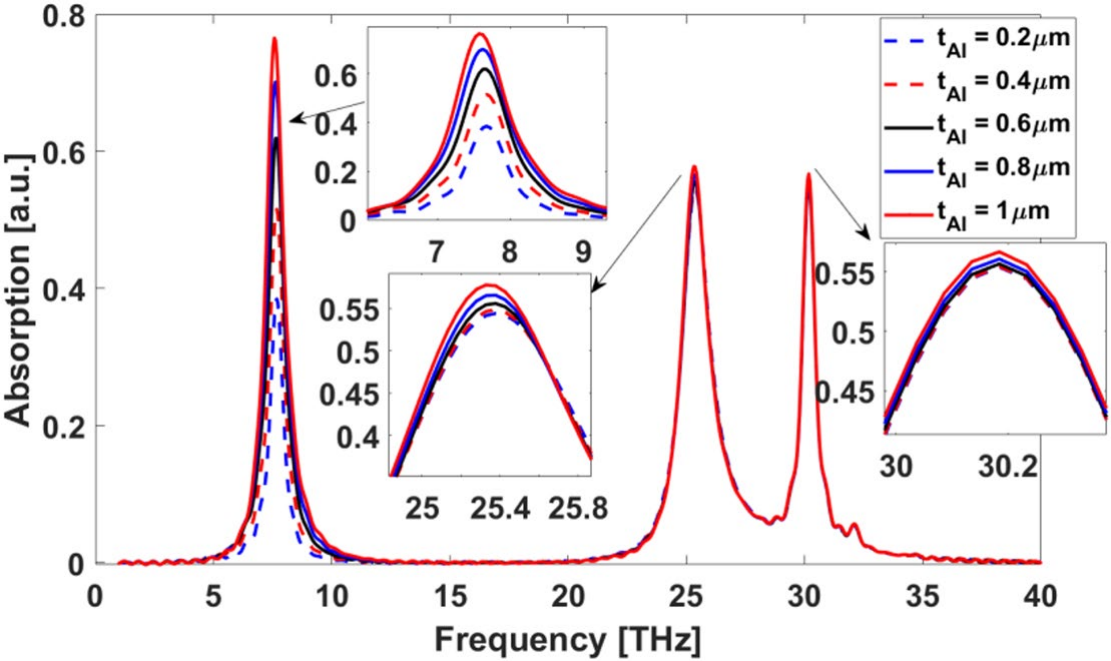
Absorption of the proposed structure for varying $\:{t}_{Al}$ from 0.2 μm to 1 μm.

The optimization of the Al layer thickness is crucial for achieving optimal absorption characteristics in the proposed structure. Figure 14 illustrates the absorption spectra for varying Al layer thicknesses $\:{t}_{Al}$ ranging from 0.2 μm to 1 μm. As observed, the absorption efficiency is highly dependent on $\:{t}_{Al}$ As the thickness increases, a clear enhancement in absorption is observed, with the 1 μm layer exhibiting the highest absorption efficiency across the studied frequency range. This improvement can be attributed to the increased interaction of incident waves with the thicker metallic layer, resulting in enhanced energy dissipation. When the ground metallic layer in the MDM absorber is dense, it effectively suppresses light transmission through the structure, resulting in enhanced optical absorption. The thick metallic substrate acts as a perfect reflector, ensuring that all incident light interacts with the upper plasmonic layer and the dielectric spacer rather than being transmitted or lost. This configuration promotes constructive interference between the incident and reflected waves within the dielectric cavity, maximizing field confinement and plasmonic resonance coupling at the metal-dielectric interfaces^50,51^. Based on these results, we select $\:{t}_{Al}$ = 1 μm as the optimal thickness, as it ensures maximum absorption while maintaining structural feasibility.

### Optimization of the silicon layer

**Fig. 15:**
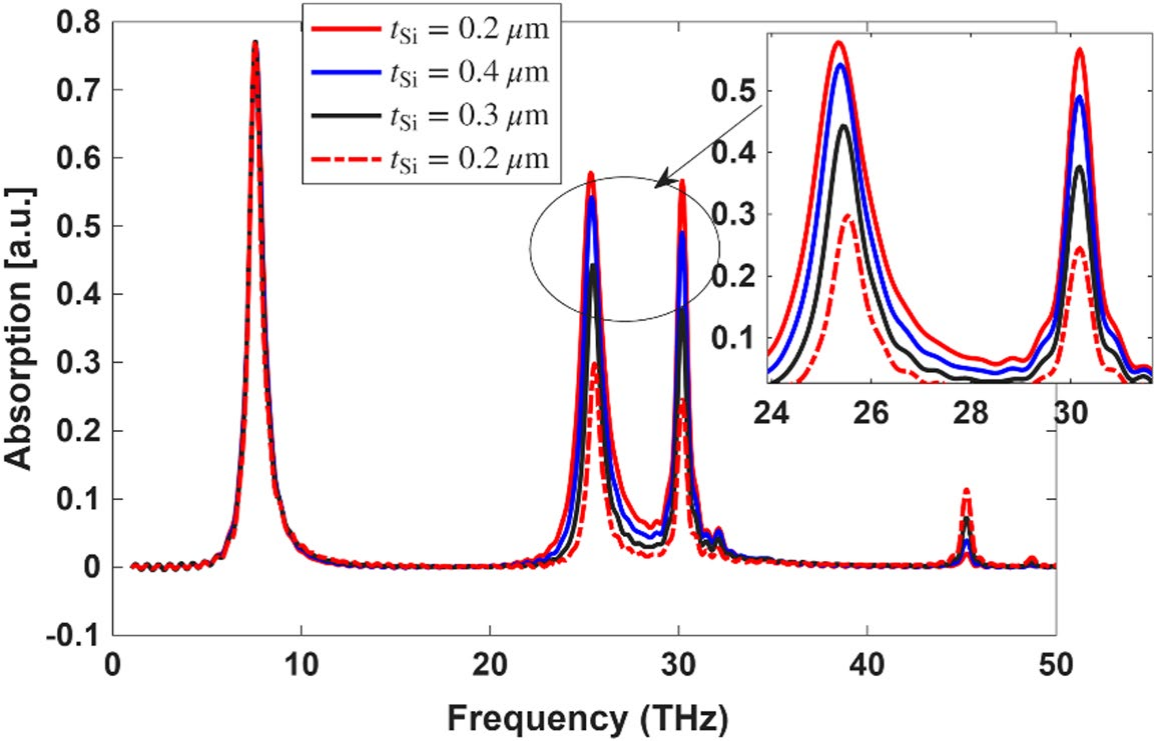
Absorption spectra versus frequency for varying $\:{t}_{Si}$ from 0.2 to 0.5 μm.

To investigate the impact of the silicon layer thickness on absorption performance, we analyzed different thicknesses ranging from 0.2 μm to 0.5 μm. As shown in Fig. 15, increasing the silicon thickness leads to noticeable changes in the absorption spectrum, particularly in the higher frequency range between 24 and 34 THz, where sharper and more intense resonance peaks are observed. The enhancement in absorption becomes more significant as the silicon layer becomes thicker, with the 0.5 μm case exhibiting the highest absorption at the key resonance frequencies. This behavior can be attributed to improved optical confinement and interaction with the resonant modes as the silicon thickness increases. Based on this analysis, we selected 0.5 μm as the optimized thickness for the silicon layer to achieve superior absorption efficiency.

## Results and discussion

**Fig. 16:**
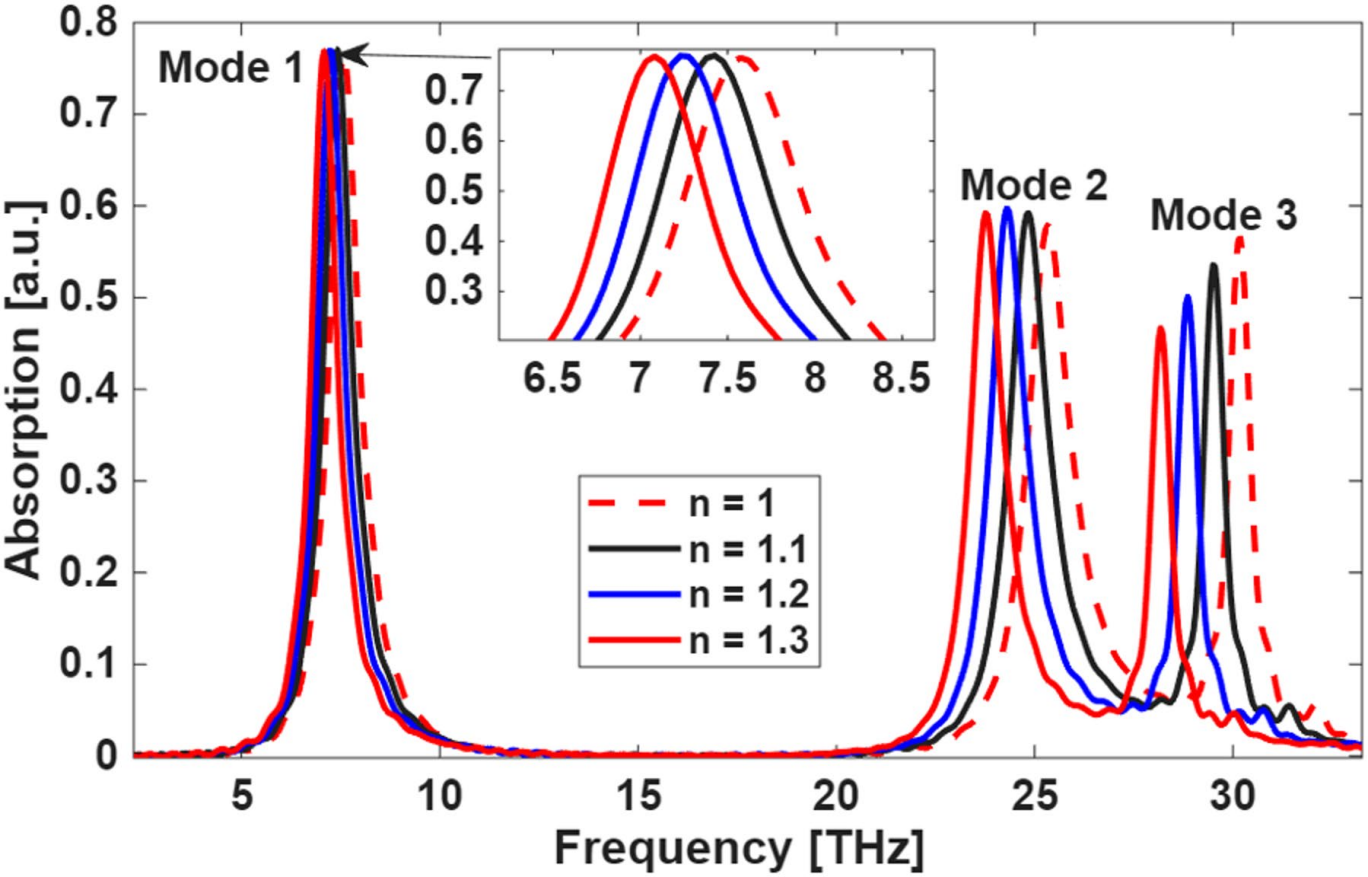
Absorption spectra of the optimized biosensor under varying refractive indices (*n* = 1.0, 1.1, 1.2, and 1.3). The sensor exhibits three characteristic peaks with different sensitivities.

After optimizing the structural parameters in Table 2, we evaluated the biosensing performance by analyzing the absorption response under varying refractive indices (*n* = 1.0 to 1.3). As shown in Fig. 16, the sensor exhibits three distinct resonant peaks, each demonstrating notable sensitivity to refractive index changes. The primary peak achieves an exceptionally high sensitivity of 10 μm/RIU, while the secondary peaks show sensitivities of 3 μm/RIU and 2.75 μm/RIU, respectively. This multi-resonance behavior not only enhances detection accuracy but also provides flexibility in monitoring different biomolecular interactions. The results confirm that the optimized design achieves high-performance biosensing, with the main peak’s sensitivity being particularly competitive for label-free detection applications. The combination of these features makes the proposed sensor a promising platform for real-time, high-precision biomedical diagnostics.

**Table 2:** Parameters optimization for the proposed biosensor.

Parameter	Value	Parameter	Value
$\:{R}_{in}$	170 nm	$\:{R}_{out}$	210 nm
$\:{r}_{i}$	12 nm	$\:{r}_{o}$	14 nm
$\:{D}_{h}$	40 nm	$\:{R}_{h}$	80 nm
$\:{t}_{Si}$	500 nm	$\:{t}_{Al}$	1 μm

**Fig. 17:**
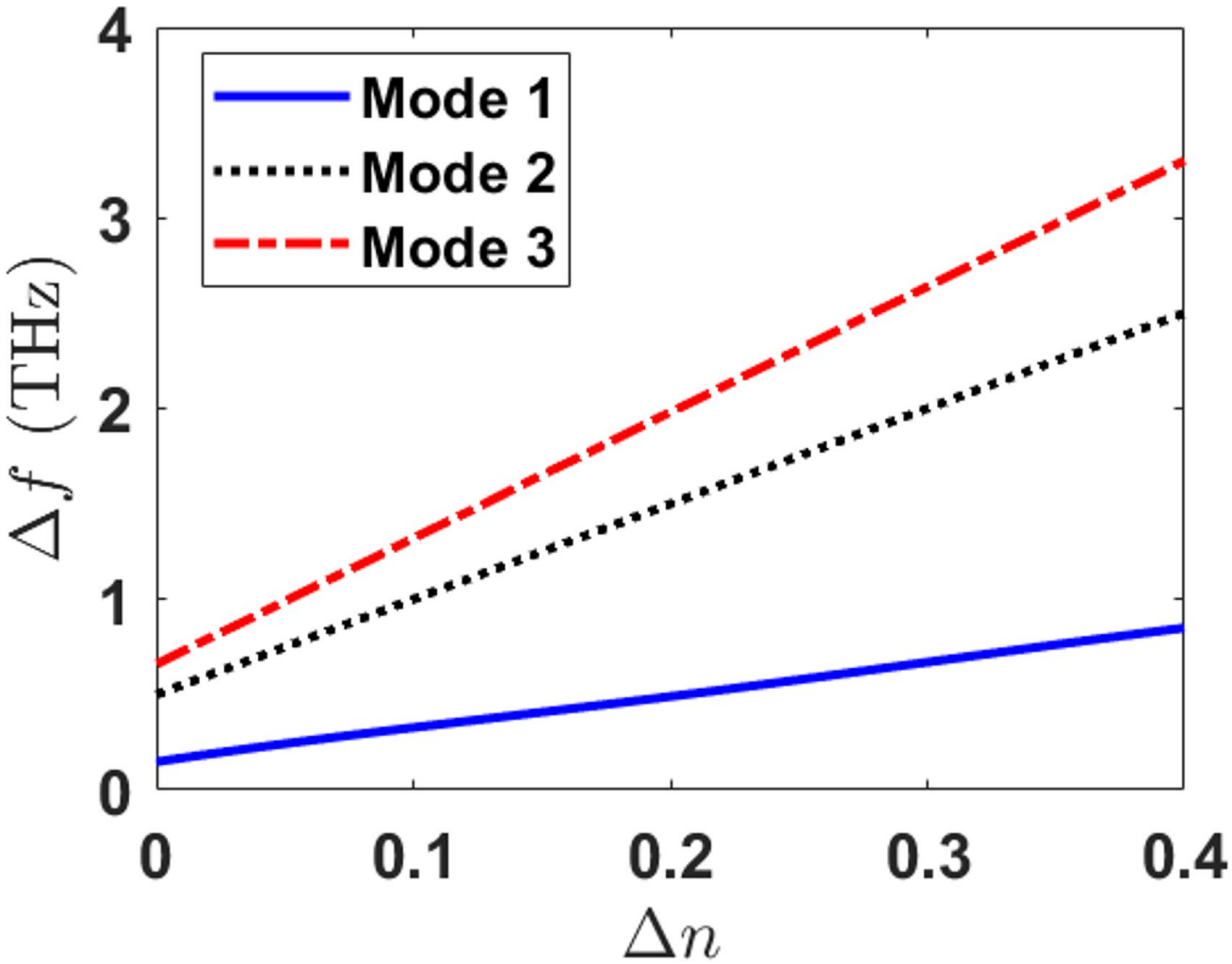
Frequency shift ($\:\varDelta\:f$) versus refractive index change ($\:n$) for the three resonant modes.

The sensor’s response to refractive index variations was further characterized by analyzing the relationship between frequency shifts and $\:n$ changes across the three resonant modes. As demonstrated in Fig. 17, all three bands exhibit a linear correlation between the frequency shift ($\:\varDelta\:f$) and refractive index ($\:\varDelta\:n)$ variation, confirming the sensor’s reliable and predictable detection capability. The linear response is particularly advantageous for quantitative biosensing applications, as it enables straightforward calibration and precise RI measurements.

The FWHM values, Q-factors, and FOMs for the three modes are listed in Table 3. For the proposed MDDM sensor, the FWHM values are 0.9 THz, 1.6 THz, and 0.1 THz for Modes 1, 2, and 3, respectively. These correspond to Q-factors of 7.66, 15.88, and 302, confirming the presence of sharp and well-defined resonances, especially in Mode 3, where the linewidth is extremely narrow. The corresponding FOMs are calculated as 11.11 RIU^−1^, 1.875 RIU^−1^, and 27.50 RIU^−1^ for Modes 1, 2, and 3, respectively. These observations indicate that the reported high sensitivities originate from spectrally narrow and high-quality resonances.

**Table 3:** Calculated resonance frequency, FWHM, Q-factor, and FOM for the three modes of the optimized MDDM sensor.

Mode	Resonance (THz)	FWHM (THz)	Q-factor	FOM (RIU^−1^)
Mode 1	7.69	0.9	7.66	11.11
Mode 2	25.40	1.6	15.875	1.875
Mode 3	30.20	0.1	302	27.50

Table 4 presents a comparative analysis between our proposed sensor and previously reported designs. In these configurations, “M” denotes metal and “D” represents dielectric materials. While earlier works have primarily focused on single-band sensing structures, such as plasmonic designs^42^, MDM (Metal–Dielectric–Metal)^52^, and MMDD (Metal–Metal–Dielectric–Dielectric)^53^. The study presented in^54^, demonstrates a four-mode plasmonic sensor that utilizes gold for an MDM, as the sensing material, achieving high sensitivity across its operational bands. Our proposed MDDM (Metal–Dielectric–Dielectric–Metal) structure offers a distinct advantage by enabling triple-band sensing. As shown in Table 4, our sensor achieves sensitivities of 10 μm/RIU, 3 μm/RIU, and 2.75 μm/RIU across three different bands, outperforming several single-band designs in terms of multi-band operation and competitive sensitivity. Furthermore, in contrast to previous structures that provide either single-band or dual-band sensing, our proposed design exhibits three well-separated resonance modes with higher FOM values (11.11 RIU^−1^, 1.875 RIU^−1^, and 27.50 RIU^−1^), demonstrating enhanced spectral sharpness and sensing precision. The integration of triple-band functionality, enhanced FOM performance, and reduced fabrication cost emphasizes the unique advantages of the proposed MDDM architecture. This makes our design a promising candidate for advanced multi-analyte detection and integrated sensing applications.

**Table 4:** A comparative analysis of the sensitivity of the proposed structure relative to reported literature results.

Ref.	Structure	Modes	Resonance (THz)	FOM (RIU^−1^)	Sensitivity (µm/RIU)	Cost
^42^	Plasmonic	Dual	16.9 and 10.6	-	2.97 and 5.20	Low
^55^	Plasmonic	Single	33.3	-	5.23 × 10^3^	Moderate
^56^	MDM	Single	0.5	-	-	High
^57^	Plasmonic	Single	4	3.16	15.59	High
^58^	MDM	Single	2.95	5.71	0.8	Moderate
^52^	MDM	Single	9.9	5.82	9.59	Moderate
^53^	MMDD	Single	333	-	2.571	Moderate
^54^	MDM	Four modes	4.32,7.48, 9.15, and 10.41	3.5, 3.47, 5.17, and 5.36	0.000913, 0.00155, 0.00196, and 0.002137	Moderate
Our work	MDDM	Trible modes	7.69, 25.40, and 30.20	11.11, 1.875, and 27.50	10, 3, and 2.75	Low

## Suggested fabrication steps

**Fig. 18:**
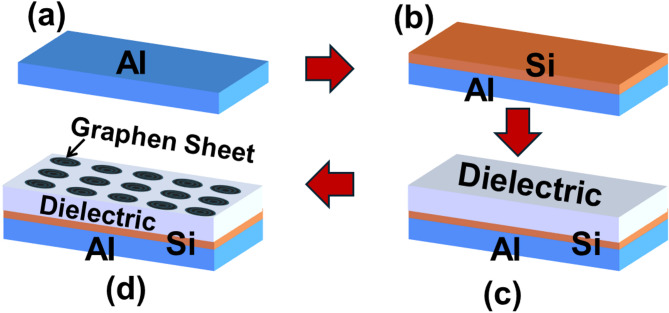
Scheme diagram of the fabrication techniques of the proposed THz sensor.

The fabrication of high-quality aluminum thin films is crucial for the performance of THz sensors, as in Fig. 18. In the process of electron-beam evaporation, a focused electron beam vaporizes ultra-pure aluminum in a vacuum, allowing for the deposition of a highly directional and contamination-free film with precise control over thickness, as in Fig. 18a. This method produces films with excellent purity, making it ideal for optical and high-frequency applications^59^. One potential method involves synthesizing high-quality monolayer graphene using chemical vapor deposition (CVD). To enhance plasmonic resonance and optimize charge transport, a periodic graphene nanomesh structure can be patterned using electron-beam lithography (EBL) and oxygen plasma etching, as shown in Fig. 18b^60^. For depositing the silicon layer in your THz sensor, Plasma-enhanced chemical vapor deposition (PECVD) is generally preferred over sputtering for high-performance applications. PECVD creates hydrogenated amorphous silicon (a-Si: H) films at moderate temperatures (200–300 °C) and offers excellent uniformity, control over the constituent materials, conformal coverage, and defect passivation, as in Fig. 18c^61^.

To achieve a high-quality dielectric layer with a $\:n$ of approximately 2 for THz sensing applications, as shown in Fig. 18d, Ion beam sputtering (IBS) is often the preferred method. IBS produces exceptionally dense films with ultra-low surface roughness (around 1 Å) and provides precise control over the refractive index, which enhances THz transparency and interfacial adhesion. A faster alternative is Ion-Assisted Electron-Beam Evaporation. This technique incorporates low-energy ions during the deposition process to densify the film, thus balancing performance with quicker growth. For ultimate precision and conformal coverage, particularly on patterned surfaces, atomic layer deposition (ALD) is ideal. ALD allows for atomic-scale thickness control and enables customization of the refractive index through layer composition design^62^.

Although fabrication is feasible, several practical challenges must be addressed when implementing the device experimentally. The initial challenge arises from defects during graphene transfer, such as wrinkles and cracks, which can reduce plasmonic performance. Dry-transfer approaches and post-transfer thermal annealing are effective methods to reduce these issues^63^. A second challenge concerns lithography alignment tolerances when patterning the metasurface above multiple layers; however, modern EBL systems routinely achieve overlay accuracies below 10 nm, and etched alignment marks further improve repeatability^64^. Finally, the narrow FWHM observed in Mode 3 (0.1 THz) makes the design sensitive to surface contamination. Thin encapsulation layers can stabilize the surface and preserve graphene quality^65^.

## Conclusion

This study presents a highly sensitive and tunable plasmonic refractive index sensor based on an MDDM metasurface structure operating in the Terahertz (THz) range. By integrating a graphene-based fractal design with metal and dielectric layers, the sensor achieves triple-band resonance with notable sensitivities of 10 μm/RIU, 3 μm/RIU, and 2.75 μm/RIU. These multiple resonant modes enhance the sensor’s capability for simultaneous or wide-range analyte detection. The robust and adaptable performance under varying environmental conditions highlights the sensor’s suitability for practical applications, including biomedical diagnostics, glucose monitoring, and gas detection. Overall, the proposed design demonstrates the significant potential of THz plasmonic metasurfaces for next-generation refractive index sensing technologies.

## Data Availability

The datasets used and/or analyzed during the current study are available from the corresponding author on reasonable request.
